# Intradermal Tranexamic Acid Injections: A Potential Therapy for Complex Cases of Drug‐Induced Postinflammatory Hyperpigmentation

**DOI:** 10.1111/jocd.70061

**Published:** 2025-02-12

**Authors:** Juan He, Shinan Hou, Yi Yang

**Affiliations:** ^1^ Medical College of Nankai University Tianjin China; ^2^ Department of Dermatology The First Medical Center of the Chinese PLA General Hospital Beijing China; ^3^ Department of Dermatology The Third Medical Center of the Chinese PLA General Hospital Beijing China

**Keywords:** efficacy, fixed drug eruption, postinflammatory hyperpigmentation, tranexamic acid, TXA

To the editor,

Generally, postinflammatory hyperpigmentation (PIH) from fixed drug eruptions (FDE) can resolve spontaneously [1]. However, various underlying factors may impede this process. Here, we present a complex patient with severe perioral hyperpigmentation due to FDE, who showed marked improvement after six sessions of intradermal tranexamic acid (TXA) injections.

A 50‐year‐old female patient presented with a 1‐year persistent hyperpigmentation on her lips and perioral area after repeatedly taking Ibuprofen to manage fever during the COVID‐19 pandemic. The pigmentation worsened with continued use of the medication (Figure 1A). Despite discontinuing ibuprofen and using topical hydroquinone and tretinoin for over 6 months, her hyperpigmentation became even more severe. A diagnosis of PIH caused by FDE was made. After discussing with her, we advised strict sun protection and avoidance of physical irritation of the lips. Additionally, we introduced intradermal injections of TXA due to its anti‐inflammatory and anti‐melanin‐producing properties. The TXA solution was prepared by diluting 0.2 mL of a 500 mg/5 mL ampoule in sterile water to a 10 mg/mL concentration, yielding a total of 2 mL. The injections were administered intradermally at a 15°–20° angle, targeting the superficial dermis and creating small semicircular wheals. During each session, 0.1 mL doses were injected 5 mm apart across 14 sites, totaling 1.4 mL per session. After six monthly TXA sessions, the patient's perioral hyperpigmentation showed significant improvement (Figure 1B).

**FIGURE 1:**
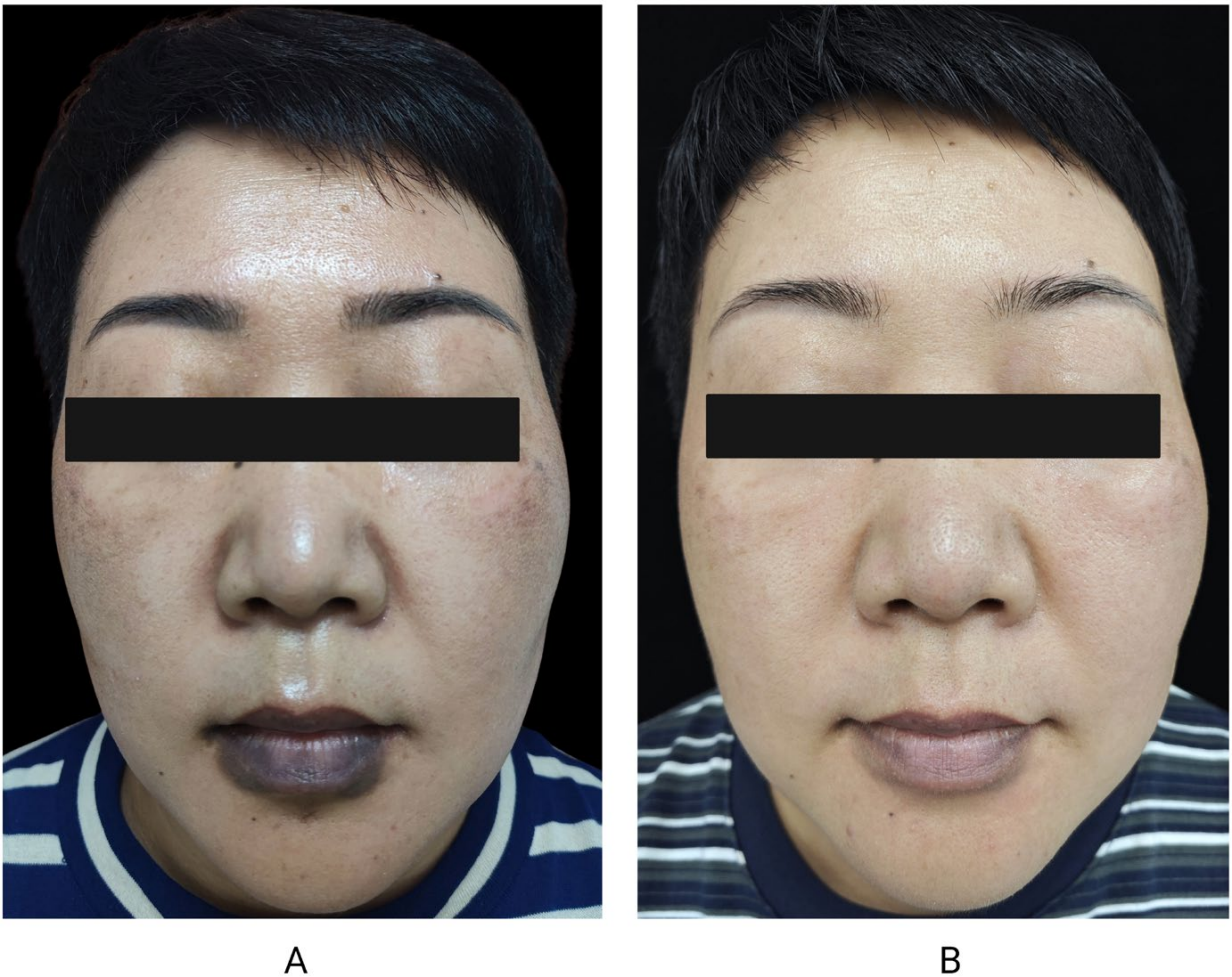
Clinical manifestation before and after treatment. (A) Noticeable hyperpigmentation on the lips and perioral area. (B) Significant reduction in lips and perioral hyperpigmentation after fix sessions of TXA intradermal injections.

The type IV hypersensitivity reaction caused by FDE leads to damage to keratinocytes and melanocytes. Following drug withdrawal, dermal macrophages then phagocytize extravasated melanin, causing persistent PIH [1]. Basically, PIH from FDE is self‐limiting [1]. However, in this case, the patient's hyperpigmentation not only failed to resolve but also worsened progressively after treatment. Hence, we considered there were additional factors that contributed to her atypical progression. First, her lesion is located on the lip, an area exposed to UV radiation and friction from eating. Second, she was in her perimenopausal period, also with skin type IV—a combination of skin type and fluctuating hormones that likely exacerbated her hyperpigmentation. Third, we can find that she also had melasma (Figure 1A), indicating a preexisting pigment metabolism abnormality. Collectively, factors such as UV exposure, mechanical friction, skin type, hormonal changes, and pigment metabolism issues likely intensified the pigmentation [2]. Therefore, for this patient, discontinuing the offending drug alone was insufficient; effective anti‐inflammatory and antipigment treatments were necessary to counteract these ongoing factors contributing to persistent hyperpigmentation.

Topical treatments, such as retinoids, hydroxy acids, and broad‐spectrum photoprotection, are the first‐line therapy for PIH [3]. Additional interventions, including oral TXA, isotretinoin, and light or laser therapies, may also be effective [3]. However, initial treatments with hydroquinone and tacrolimus had worsened her condition, likely due to their irritative effects on this delicate region. Considering that the hyperpigmentation centered on the lip mucosa, a sensitive area prone to irritation, we opted for intradermal TXA injections. TXA is a synthetic lysine analog that inhibits fibrinolysis, stabilizes blood clots, and can reduce inflammation [4]. Studies have revealed that TXA can also prevent melanocyte activation induced by UV light, hormones, and injured keratinocytes by inhibiting the plasminogen activator system [4]. Owing to its anti‐inflammatory and anti‐melanogenic properties, TXA has been used for PIH, melasma, and other cutaneous inflammatory conditions [5]. Besides, studies indicate that intradermal TXA is the most effective and cost‐efficient option, providing greater efficacy and fewer side effects compared to oral or topical forms [6]. In this case, breaking the loop between pigmentation and the underlying inflammatory response is crucial for the resolution of pigmentation.

For drug‐induced PIH, a comprehensive analysis should identify factors hindering pigmentation recovery, and treatments should be tailored to each patient. In this case, we have observed that intradermal TXA injections may represent a promising therapeutic option for patients with PIH complicated by underlying inflammatory responses, owing to their dual anti‐inflammatory and antipigmentary properties.

## Conflicts of Interest

The authors declare no conflicts of interest.

## Data Availability

The data that support the findings of this study are available on request from the corresponding author. The data are not publicly available due to privacy or ethical restrictions.
